# Effects of sources and levels of dietary supplementary manganese on growing yak’s *in vitro* rumen fermentation

**DOI:** 10.3389/fvets.2023.1175894

**Published:** 2023-06-09

**Authors:** Huizhen Lu, Pengpeng Liu, Shujie Liu, Xinsheng Zhao, Binqiang Bai, Jianbo Cheng, Zijun Zhang, Cai Sun, Lizhuang Hao, Yanfeng Xue

**Affiliations:** ^1^Department of Animal Science, College of Animal Science and Technology, Anhui Agricultural University, Hefei, China; ^2^Biotechnology Center, Anhui Agricultural University, Hefei, China; ^3^Qinghai Pure Yak Biotechnology Co., LTD., Xining, China; ^4^State Key Laboratory of Plateau Ecology and Agriculture, Key Laboratory of Plateau Grazing Animal Nutrition and Feed Science of Qinghai Province, Qinghai Plateau Yak Research Center, Qinghai Academy of Science and Veterinary Medicine, Qinghai University, Xining, China

**Keywords:** manganese, rumen fermentation *in vitro*, digestive enzyme activities, volatile fatty acids, microbial protein

## Abstract

**Introduction:**

Manganese (Mn) is an essential trace element for livestock, but little is known about the optimal Mn source and level for yak.

**Methods:**

To improve yak’s feeding standards, a 48-h *in vitro* study was designed to examine the effect of supplementary Mn sources including Mn sulfate (MnSO_4_), Mn chloride (MnCl_2_), and Mn methionine (Met-Mn) at five Mn levels, namely 35 mg/kg, 40 mg/kg, 50 mg/kg, 60 mg/kg, and 70 mg/kg dry matter (includes Mn in substrates), on yak’s rumen fermentation.

**Results:**

Results showed that Met-Mn groups showed higher acetate (*p* < 0.05), propionate, total volatile fatty acids (*p* < 0.05) levels, ammonia nitrogen concentration (*p* < 0.05), dry matter digestibility (DMD), and amylase activities (*p* < 0.05) compared to MnSO4 and MnCl2 groups. DMD (*p* < 0.05), amylase activities, and trypsin activities (*p* < 0.05) all increased firstly and then decreased with the increase of Mn level and reached high values at 40–50 mg/kg Mn levels. Cellulase activities showed high values (*p* < 0.05) at 50–70 mg/kg Mn levels. Microbial protein contents (*p* < 0.05) and lipase activities of Mn-Met groups were higher than those of MnSO4 and MnCl2 groups at 40–50 mg/kg Mn levels.

**Discussion:**

Therefore, Mn-met was the best Mn source, and 40 to 50 mg/kg was the best Mn level for rumen fermentation of yaks.

## 1. Introduction

Yak (*Poephagus grunniens*) is a kind of domestic animal who lives on the Asian plateau between 3,000 and 5,000 meters above sea level (1). Local herdsmen mainly rely on grazing on natural pasture to raise yaks (2), so nutrient intakes of yaks depend heavily on the forage supply. However, forage supply on the Qinghai-Tibet plateau varied with time, which is abundant in the warm season from May to September but extremely deficient in the cold season from October to April (3). Rational feed supplementation can improve the growth performance of yaks (4). However, there is not a good feeding standard for yaks due to the lack of knowledge about mineral requirements. There is an increasing evidence that minerals are important for the health of herbivores (5) since they are key cofactors and components of enzymes in various biological processes (6). Yak’s mineral sources are mainly pasture and shrubs, and sometimes small amounts of minerals are taken from the soil (7, 8). However, pasture in most areas cannot meet the mineral requirements of grazing animals (9) and grazing yaks in the Qinghai-Tibet plateau are deficient in minerals (10). Mineral deficiency can impair growth and reproduction of animals and damage immune function and even lead to death (11).

Manganese (Mn) is an essential trace element and plays an important role in growth, development, and metabolism (12). Mn is an activator of two important enzymes necessary for the synthesis of chondroitin sulfate: polysaccharide polymerase and galactose transferase (13, 14). *In vitro* culture experiments show that Mn deficiency reduces cellulose digestibility and Mn supplementation improves cellulose digestibility and dry matter digestibility (DMD) (15, 16), whereas, extremely high dose of Mn (300 ppm) inhibits cellulose digestion (16). Moreover, Mn is one of the trace elements essential for certain rumen microorganisms (17). In male lambs, higher Mn intake increases the proportion of large rumen bacteria (18). However, the study of Inner Mongolia grassland of China finds that plants are seriously lacking in Mn, iron, copper, and zinc for ruminants (19). Further, Mn content of various silages is lower than the concentration indicated in the feed ingredient list (20) and lower than the requirement of dairy cows (21). Additionally, diets with high calcium, phosphorus, and iron levels also reduce the availability of Mn (20). In general, Mn is usually supplemented in the form of inorganic Mn such as Mn oxide, Mn chloride (MnCl_2_), and Mn sulfate (MnSO_4_) (22) and organic Mn such as Mn-methionine (Mn-Met), Mn-proteinate, and Mn-polysaccharide (23). NRC (1996) (24) recommends 20–40 mg/kg Mn for beef cattle. However, Mn requirement for growing yaks is still unclear.

Therefore, to maximize the positive effects of Mn in yak breeding, it’s necessary to formulate a reasonable Mn supplement scheme and select an appropriate Mn supplemental type and level. Rumen is an essential fermentation tank for the preparation of fermentation end products, especially volatile fatty acids (VFAs) and microbial protein (MCP), which are the main energy and protein sources for the ruminants (25). The higher the rumen efficiency, the better the fermentation end product synthesis (25), which indirectly reflects the productivity of ruminants. This study aimed to dissect the effects of different sources and levels of Mn on rumen fermentation of yaks through *in vitro* fermentation, and finally provided data support for the reasonable supplementation of Mn in yak feeding.

## 2. Materials and methods

### 2.1. Laboratory animals and feeding management

Animal experiments were conducted in accordance with the Guidelines for the Conservation and Utilization of Laboratory Animals of Qinghai Province (Qinghai Bureau of Agriculture and Animal Husbandry, 2002) and were approved by the Professional Committee of Animal Use, Academy of Animal Husbandry and Veterinary Medicine in Qinghai University (QHU20150301). According to the requirements of Chinese Beef Cattle Breeding (NY/T815-2004) (26) and Yak Nutrition Monograph (27), three healthy Datong yaks with permanent rumen fistulas were fed 4 kg/day diet. The ingredients and nutritional levels of the diet were shown in Table 1. Yaks were fed the in the individual pens twice a day (8:00 and 18:00) and had free access to water.

**Table 1:** Formula of basic ration and conventional nutrient levels (dry matter basis).

Item	%	Component	%
Corn flour	36.00	Moisture	9.02
Wheat bran	12.00	Crude protein	11.03
Soybean meal	6.60	Acid detergent fiber	17.60
Rapeseed meal	2.13	Neutral detergent fiber	31.12
Stone powder	0.90	Ca	0.88
CaHPO_4_	0.17	P	0.19
NaCl	0.58	Mn (mg/kg)	33.819
Oat hay	40.00		

### 2.2. Determination of Mn content

According to the method described in GB/T13885-2003 (28), 5 mg diet sample was carbonized in a crucible and completely incinerated in a muffle furnace (SX-4-10, Taisite Instrument Company, Tianjin, China) at 550°C for 4 h. The ash was cooled to room temperature and dissolved in 5 mL of 6 mol/L HCl, which was then diluted to 50 mL using a volumetric flask. Subsequently, an atomic absorption spectrophotometer (TAS-990, Beijing Puxi General Instrument Company, Beijing, China) was used to determine the content of trace element Mn in the dilution (29). The parameters for the determination of Mn content in the diet were set as follows: wavelength 279.5 nm, slit width 0.2 nm, lighting mode NON-BGC, minimum lamp current 10 mA. Finally, we obtained the Mn content in the diet was 33.819 mg/kg.

### 2.3. Experimental design

A 3 × 5 two-factor design with 3 Mn types and 5 Mn levels was adopted for the fermentation *in vitro*. The 3 Mn types were MnSO_4_, MnCl_2_, and Met-Mn (Xingjia Bioengineering Company, Changsha, China). We used the diet as substrate for fermentation *in vitro*, so Mn content of the basic fermented substrate was also 33.819 mg/kg. Mn sources were added into the fermented substrates to make the final Mn contents reach 35 mg/kg, 40 mg/kg, 50 mg/kg, 60 mg/kg, and 70 mg/kg, respectively.

### 2.4. Yak’s rumen fermentation *in vitro*

After 15 days of adaption period, yak’s rumen fluid samples were collected from the fistulas before morning feeding using a vacuum pump (2 XZ-1, Kewei Yongxing Instrument Company, Beijing, China), mixed well, and brought back to the laboratory. After filtration with four layers of gauze, the rumen fluid was kept warm for fermentation *in vitro* later. Artificial fermentation solutions were prepared according to the formula in Table 2 (30). Then, 667 mL pure water, 0.17 mL trace elements solution A, 333 mL buffer solution B, 333 mL macro element solution C, and 1.7 mL resazurin indicator solution, and 67 mL deoxidizer solution were, successively, added into a 2000 mL glass jar warmed in water bath at 39°C. A magnetic stirrer was used to mix the solutions and CO_2_ was continuously injected into the mixed solution until it faded and become colorless. Subsequently, 750 mL rumen fluid was added into the mixed solution (31) and CO_2_ was injected for another 30 min to remove the dissolved air, during which the fermentation fluid was kept in a water bath at 39°C to ensure the activity of rumen microorganisms.

**Table 2:** Each single solution’s formula of artificial rumen buffer.

Micro element solution (A liquid)	Buffer (B liquid)	Macro element solution (C liquid)	Indicator solution	Deoxidizer solution
CaCl_2_·2H_2_O 6.60 g CoCl_2_·6H_2_O 0.50 g FeCl_3_·6H_2_O 4.00 g	NH_4_HCO_3_ 0.80 g NaHCO_3_ 7.00 g	Na_2_HPO_4_ 1.14 g KH_2_PO_4_ 1.24 g MgSO_4_·7H_2_O 0.12 g	resazurin 100 mg	NaOH 0.16 g Na_2_S·9H_2_O 625.0 mg
Add ultrapure water to 50 mL	Add ultrapure water to 200 mL	Add ultrapure water to 200 mL	Add ultrapure water to 100 mL	Add ultrapure water to 100 mL

Yak’s diet was physically ground and sifted through a 40-mesh sieve (pore size 0.45 mm), which was used as the fermented substrate. Different types (MnSO_4_, MnCl_2_, and Met-Mn) and levels of Mn were added into the fermented substrates to reach the designed Mn levels 35 mg/kg, 40 mg/kg, 50 mg/kg, 60 mg/kg, and 70 mg/kg. Then, 200 mg fermentation substrate was accurately weighed using an analytical balance and loaded into a glass fermentation syringe (Fortuna Häberle Laboratory, Lonsee-Ettlenschie β, Germany) and 30 mL fermentation fluid was added to each fermentation syringe using a liquid separation device (Hirschmann-Laborgeräte, Eberstadt, Germany).

### 2.5. Determination of total gas production and methane yield

All the fermentation syringes were transferred into a preheated thermostatic artificial rumen incubator (temperature 39 ± 0.5°C, rotation speed 40 r/min) and cultured 48 h to record the total gas production (31). At the midpoint and the end point of fermentation time, the gas produced by fermentation was collected into a vacuum tube (Xinle Company, Shijiazhuang, China) for the determination of methane content in the gas. Methane content was determined using a gas chromatograph (GC-2014, Shimadzu, Kyoto, Japan) with FID detector and capillary column (FFAP30 m × 0.32 mm × 0.5 μm). The temperatures of column, vaporization chamber, and detector were set at 100°C, 100°C, and 110°C, respectively. Carrier gas was high-purity nitrogen (99.99%) with the pressure of 0.7 MPa. Hydrogen pressure and air pressure of detector were both set at 0.4 MPa while capillary column pressure was set at 65 KPa.

### 2.6. Determination of pH, NH_3_-N, MCP, DMD, VFAs, and digestive enzyme activities

After 48 h, the fermentation syringes were quickly inserted into the ice-water mixture to stop the fermentation and the pH was measured immediately using a high-precision acidometer (Hanna HI221, Ann Arbor, MI, United States). Ammonia nitrogen (NH_3_-N) concentration was measured using a spectrophotometry (TU-1810, Beijing Puxi Instrument Company, Beijing, China) according to the method described by Feng and Gao (32). MCP content was detected using a kit (A045-1-1, Nanjing Jiancheng Bioengineering Institute, Nanjing, China) according to the manufacturer’s instruction. The formula was MCP content (g/L) = (A*_m_* – A*_k_*)/(A*_s_*-A*_k_*) × C*_k_* × w, in which A*_m_*, A*_k_*, and A*_s_* were the absorption values of sample tube, blank tube, and standard tube, respectively while C*_k_* was the protein standard concentration of 56.3 g/L and w is the dilution times. The formula of DMD was DMD (%) = (sample substrate weight - sample residue weight + blank tube resides weight)/sample substrate weight × 100.

VFAs levels in the fermented fluid were determined by a Gas Chromatography (GC-2014, Shimadz, Kyoto, Japan) with FID detector and FFAP capillary column (30 m × 0.32 mm × 0.5 μm) using the method described by Zhao et al. (33). The column temperature procedure was initial 60°C, an increasing of 10°C /min to 120°C, holding at 120°C for 3 min, an increasing of 15°C /min to 180°C, and holding at 180°C for 5 min. Both the temperatures of vaporization chamber and detector were set at 250°C. Carrier gas was high-purity nitrogen (99.99%) with the pressure of 0.7 MPa. Hydrogen pressure and air pressure of detector were both set at 0.4 MPa while capillary column pressure was set at 0.6–0.8 MPa. Digestive enzyme activities in the fermented fluid including amylase, lipase, trypsin, and cellulose were detected using commercial amylase kit (C016-1-1), lipase kit (A054-1-1), trypsin kit (A080-2-1), and cellulose kit (A138-1-1), respectively, from Nanjing Jiancheng Bioengineering Institute according to the manufacturer’s instructions (33).

### 2.7. Statistical analysis

Two-way ANOVA was performed using SPSS 19.0 (SPSS Inc., Chicago, IL, United States) software to analyze the effects of general Mn type, Mn level, and the Mn type & level interaction on fermentation parameters. When the Mn type & level interaction was significant, one-way ANOVA was used to make multiple comparisons among different Mn level groups with the same Mn type and among different Mn type groups at the same Mn level. When the Mn type & Mn level interaction was not significant, Duncan’s range test was used to do multiple comparisons among different Mn type groups (without regard to different Mn levels) and among different Mn level groups (without regard to different Mn types). The orthogonal polynomial contrast method was used to determine whether the effect of Mn on the measured variable is linear or quadratic with the increase of Mn level. All data were expressed as Mean ± SD. *p* < 0.05 was used to judge significant difference.

## 3. Results

### 3.1. Gas production *in vitro*

Mn level & type interaction (*p* = 0.006) had a significant effect on total gas production (Table 3). For different Mn levels, when MnCl_2_ was used as a Mn source, the total gas production in the 50 mg/kg Mn level group was significantly higher than all the other groups (*p* < 0.05); when MnSO_4_ or Met-Mn was used as a Mn source, no significant difference (*p* > 0.05) was observed among different Mn level groups (Table 3). For different Mn types, at 50 mg/kg Mn level, the total gas production of MnCl_2_ group was significantly higher than those of MnSO_4_ and Met-Mn groups, and at 70 mg/kg Mn level, the total gas production of MnCl_2_ group was significantly higher than that of Met-Mn group (*p* < 0.05, Table 3). Mn source (*p* = 0.46), Mn level (*p* = 0.756), or Mn level & type interaction (*p* = 0.621) had no significant effect on methane production (Table 3).

**Table 3:** *In vitro* total gas and methane yield from substrate with added manganese (Mn) from different sources at different levels^1^.

Mn Source	Mn (mg/kg DM)^2^	Total gas production (mL)	Methane yield (mL)
MnSO_4_	35 mg/kg	70.0 ± 3.6^A^	5.1 ± 2.8
	40 mg/kg	64.2 ± 3.8	4.5 ± 1.6
	50 mg/kg	67.3 ± 0.8^B^	4.8 ± 1.6
	60 mg/kg	67.7 ± 4.6	6.1 ± 0.8
	70 mg/kg	60.0 ± 6.2^AB^	5.6 ± 0.6
*p*-value	Treat	0.106	–
	Linear	0.057	–
	Quadratic	0.491	–
MnCl_2_	35 mg/kg	67.3 ± 0.3^bAB^	5.5 ± 0.7
	40 mg/kg	63.0 ± 1.0^c^	5.1 ± 1.5
	50 mg/kg	70.8 ± 1.3^aA^	5.2 ± 3.0
	60 mg/kg	61.3 ± 1.8^c^	6.8 ± 1.4
	70 mg/kg	67.8 ± 1.9^bA^	5.5 ± 2.6
*p*-value	Treat	0.000	–
	Linear	0.814	–
	Quadratic	0.166	–
Mn-Met	35 mg/kg	59.0 ± 6.3^B^	5.7 ± 0.7
	40 mg/kg	65.0 ± 2.5	5.4 ± 0.1
	50 mg/kg	64.3 ± 0.8^C^	7.3 ± 0.6
	60 mg/kg	64.2 ± 5.8	4.9 ± 1.5
	70 mg/kg	56.3 ± 4.3^B^	6.4 ± 0.1
*p*-value	Treat	0.133	–
	Linear	0.464	–
	Quadratic	0.018	–
Source	MnSO_4_	65.8 ± 5	5.2 ± 1.5
	MnCl_2_	66.0 ± 3.7	5.6 ± 1.8
	Mn-Met	61.8 ± 5.2	5.9 ± 1.1
Level	35 mg/kg	65.4 ± 6.1	5.4 ± 1.5
	40 mg/kg	64.1 ± 2.5	5.0 ± 1.2
	50 mg/kg	67.4 ± 2.9	5.7 ± 2.1
	60 mg/kg	64.3 ± 4.7	5.9 ± 1.4
	70 mg/kg	61.4 ± 6.4	5.8 ± 1.4
*p*-value	Linear	–	–
	Quadratic	–	–
*p*-value	Source	–	0.460
	Level	–	0.756
	S × L^3^	0.006	0.621

1 Different lowercase letters indicate significant differences in the means of each variable between different Mn levels at the same Mn types (*p* < 0.05), and different uppercase letters indicate significant differences in the means of each variable between different Mn types at the same Mn level (*p* < 0.05).

2 Includes added Mn and Mn in substrate.

3 S × L: Source × Level interaction.

### 3.2. DMD and pH of fermentation fluid *in vitro*

Overall, Mn type (*p* < 0.001) had a significant effect on pH, while Mn level (*p* = 0.385) and Mn level & type interaction (*p* = 0.116) had no significant effect (Table 4). For different Mn types, the pH of MnCl_2_ group was significantly higher than the other two groups (*p* < 0.05). Both Mn type (*p* = 0.011) and level (*p* < 0.001) had significant effects on DMD but no significant (*p* = 0.326) interaction between Mn level & type (Table 4). The DMD of Met-Mn group was the highest and was significantly higher than that of MnCl_2_ group (*p* < 0.05) (Table 4). For different Mn levels, DMD showed a quadratic change trend (*p* < 0.001) of first increasing and then decreasing with the increase of Mn level and reached the peak value at 50 mg/kg Mn level. The DMD of 50 mg/kg group was significantly higher than those of 35 mg/kg, 40 mg/kg, and 60 mg/kg Mn level groups (*p* < 0.05, Table 4).

**Table 4:** *In vitro* pH and dry matter digestibility (DMD) from substrate with added manganese (Mn) from different sources at different levels^1^.

Mn Source	Mn (mg/kg DM)^2^	pH	DMD
MnSO_4_	35 mg/kg	6.9 ± 0.2	38.2 ± 4.2
	40 mg/kg	7.1 ± 0.2	55.9 ± 13.1
	50 mg/kg	7.2 ± 0.2	67.7 ± 10.1
	60 mg/kg	7.1 ± 0.1	60.8 ± 5.9
	70 mg/kg	7.0 ± 0.3	65.4 ± 11.1
*p*-value	Treat	–	–
	Linear	–	–
	Quadratic	–	–
MnCl_2_	35 mg/kg	7.3 ± 0.1	48.4 ± 5.4
	40 mg/kg	7.5 ± 0.1	54.5 ± 5.1
	50 mg/kg	7.5 ± 0.1	60.3 ± 3.5
	60 mg/kg	7.4 ± 0.1	51.4 ± 3.7
	70 mg/kg	7.4 ± 0.1	52.9 ± 3.7
*p*-value	Treat	–	–
	Linear	–	–
	Quadratic	–	–
Mn-Met	35 mg/kg	7.1 ± 0.1	48.2 ± 7.2
	40 mg/kg	6.9 ± 0.1	66.4 ± 8.7
	50 mg/kg	7.0 ± 0.	74.4 ± 9.1
	60 mg/kg	7.0 ± 0.1	63.0 ± 9.6
	70 mg/kg	7.2 ± 0.1	61.6 ± 8.3
*p*-value	Treat	–	–
	Linear	–	–
	Quadratic	–	–
Source	MnSO_4_	7.1 ± 0.2^B^	57.6 ± 13.5^AB^
	MnCl_2_	7.4 ± 0.1^A^	53.5 ± 5.5^B^
	Mn-Met	7.0 ± 0.1^B^	62.7 ± 11.4^A^
Level	35 mg/kg	7.1 ± 0.2	44.5 ± 7.1^c^
	40 mg/kg	7.1 ± 0.3	58.9 ± 10^b^
	50 mg/kg	7.2 ± 0.3	67.4 ± 9.3^a^
	60 mg/kg	7.2 ± 0.2	58.3 ± 7.9^b^
	70 mg/kg	7.2 ± 0.2	60 ± 9.1^ab^
*p*-value	Linear	–	0.001
	Quadratic	–	0.000
*p*-value	Source	0.000	0.011
	Level	0.385	0.000
	S × L^3^	0.116	0.326

1 Different lowercase letters indicate significant differences in the means of each variable between different Mn levels at the same Mn types (*P* < 0.05), and different uppercase letters indicate significant differences in the means of each variable between different Mn types at the same Mn level (*p* < 0.05).

2 Includes added Mn and Mn in substrate.

3 S × L: Source × Level interaction.

### 3.3. Digestive enzyme activity *in vitro*

Only the Mn level & type interaction was significant for lipase activity (*p* = 0.029) but not for amylase (*p* = 0.209), trypsin (*p* = 0.721), and cellulose (*p* = 0.584) activities (Table 5). When MnCl_2_ was used as a Mn source, Mn level had a significant effect on lipase activity (*p* = 0.033), and the lipase activity of 50 mg/kg group was significantly higher (*p* < 0.05) than those of 35 mg/kg, 40 mg/kg, and 70 mg/kg Mn level groups; when Mn-Met was used as a Mn source, Mn level also had a significant effect on lipase activity (*p* < 0.001), and the lipase activity of 50 mg/kg group was significantly higher (*p* < 0.05) than the other groups; when MnSO_4_ was used as a Mn source, no significant difference (*p* = 0.076) was observed among different Mn level groups (Table 5). For different Mn types, at 50 mg/kg Mn level, the lipase activities of Met-Mn and MnCl_2_ groups were both higher than that of MnSO_4_ group. Mn type had a significant effect on amylase activity (*p* < 0.001) but had no significant effect on trypsin (*p* = 0.33) and cellulose (*p* = 0.151) activities (Table 5). The amylase activity of Met-Mn group was higher than those of MnSO_4_ (*p* < 0.05) and MnCl_2_ groups (*p* < 0.05, Table 5). Mn level showed significant effect on amylase (*p* = 0.008), trypsin (*p* < 0.001), and cellulose (*p* < 0.001) activities for yak’s rumen fermentation *in vitro* (Table 5). With the increase of Mn level, the activity of trypsin increased firstly and then decreased which showed a quadratic change trend (*p* < 0.001) while the activity of cellulose linearly increased (*p* < 0.001). The activities of amylase, trypsin, and cellulose reached the high values at 40 mg/kg, 40–50 mg/kg, and 50–70 mg/kg Mn levels, respectively (Table 5). Therefore, Mn-Met was the optimal Mn type, and the optimal Mn levels for amylase, lipase, trypsin, and cellulase activities were 40 mg/kg, 50 mg/kg, 40–50 mg/kg, and 50–70 mg/kg, respectively.

**Table 5:** *In vitro* digestive enzyme activities from substrate with added manganese (Mn) from different sources at different levels^1^.

Mn Source	Mn (mg/kg DM)^2^	Amylase activity	Lipase activity	Trypsin activity	Cellulase activity
MnSO_4_	35 mg/kg	1.3 ± 0.2	0.4 ± 0.0^AB^	28.1 ± 14	53.3 ± 2.0
	40 mg/kg	1.8 ± 0.0	0.4 ± 0.0^B^	74.8 ± 8.1	68.4 ± 5.3
	50 mg/kg	1.3 ± 0.2	0.4 ± 0.0^B^	70.1 ± 14.0	78.8 ± 4.0
	60 mg/kg	1.2 ± 0.4	0.5 ± 0.1	60.8 ± 21.4	86.9 ± 7.0
	70 mg/kg	1.3 ± 0.1	0.4 ± 0.0	28.1 ± 14.0	83.4 ± 9.2
*p*-value	Treat	–	0.076	–	–
	Linear	–	0.095	–	–
	Quadratic	–	0.116	–	–
MnCl_2_	35 mg/kg	1.1 ± 0.2	0.3 ± 0.1^bB^	32.7 ± 8.1	56.8 ± 5.3
	40 mg/kg	1.2 ± 0.1	0.3 ± 0.0^bC^	65.5 ± 8.1	74.2 ± 8.7
	50 mg/kg	1.0 ± 0.4	0.5 ± 0.0^aA^	56.1 ± 14.0	84.6 ± 8.7
	60 mg/kg	0.8 ± 0.2	0.4 ± 0.0^ab^	42.1 ± 14.0	81.1 ± 8.7
	70 mg/kg	0.9 ± 0.0	0.3 ± 0.2^b^	37.4 ± 16.2	81.1 ± 11.2
*p*-value	Treat	–	0.033	–	–
	Linear	–	0.760	–	–
	Quadratic	–	0.005	–	–
Mn-Met	35 mg/kg	1.9 ± 0.0	0.4 ± 0.0^cA^	32.7 ± 8.1	67.2 ± 4.0
	40 mg/kg	2.2 ± 0.0	0.5 ± 0.0^bA^	93.5 ± 29.2	69.5 ± 9.2
	50 mg/kg	2.2 ± 0.0	0.5 ± 0.0^aA^	60.8 ± 8.1	86.9 ± 9.2
	60 mg/kg	2.1 ± 0.0	0.4 ± 0.0^c^	51.4 ± 42.9	85.8 ± 8.7
	70 mg/kg	2.1 ± 0.1	0.4 ± 0.0^d^	46.8 ± 21.4	88.1 ± 5.3
*p*-value	Treat	–	0.000	–	–
	Linear	–	0.001	–	–
	Quadratic	–	0.000	–	–
Source	MnSO_4_	1.4 ± 0.3^B^	0.4 ± 0.1	52.4 ± 24.6	74.2 ± 13.6
	MnCl_2_	1.0 ± 0.2^C^	0.4 ± 0.1	46.8 ± 16.5	75.6 ± 12.7
	Mn-Met	2.1 ± 0.1^A^	0.4 ± 0.1	57.0 ± 30.2	79.5 ± 11.4
Level	35 mg/kg	1.5 ± 0.4^b^	0.4 ± 0.1	31.1 ± 9.3^d^	59.1 ± 7.1^c^
	40 mg/kg	1.7 ± 0.5^a^	0.4 ± 0.1	77.9 ± 20.0^a^	70.7 ± 7.4^b^
	50 mg/kg	1.5 ± 0.6^b^	0.5 ± 0.1	62.3 ± 12.3^ab^	83.4 ± 7.6^a^
	60 mg/kg	1.4 ± 0.6^b^	0.5 ± 0.1	51.4 ± 26.2^bc^	84.6 ± 7.6^a^
	70 mg/kg	1.5 ± 0.5^b^	0.3 ± 0.1	37.4 ± 17.1^cd^	84.2 ± 8.3^a^
*p*-value	Linear	0.101	–	0.480	0.000
	Quadratic	0.269	–	0.000	0.001
*p*-value	Source	0.000	–	0.330	0.151
	Level	0.008	–	0.000	0.000
	S × L^3^	0.209	0.029	0.721	0.584

1 Different lowercase letters indicate significant differences in the means of each variable between different Mn levels at the same Mn types (*P* < 0.05), and different uppercase letters indicate significant differences in the means of each variable between different Mn types at the same Mn level (*p* < 0.05).

2 Includes added Mn and Mn in substrate.

3 S × L: Source × Level interaction.

### 3.4. VFAs levels in fermentation fluid *in vitro*

Mn level & type interaction had a significant effect on acetate/propionate (*p* = 0.033) but had no significant effects on acetate (*p* = 0.153), propionate (*p* = 0.122), isobutyrate (*p* = 0.65), butyrate (*p* = 0.428), isovalerate (*p* = 0.592), valerate (*p* = 0.656), and total VFAs (*p* = 0.257) levels (Table 6). For different Mn levels, acetate/propionate showed a linear increase (*p* = 0.01) with the increase of Mn levels when MnCl_2_ was used as a Mn source, and the acetate/propionate was highest at 50 mg/kg and 70 mg/kg Mn levels, which was higher than that of 35 mg/kg and 40 mg/kg Mn level groups (*p* < 0.05, Table 6); when MnSO_4_ or Met-Mn was used as a Mn source, no significant difference (*p* > 0.05) was observed among different Mn levels. For different Mn types, acetate/propionate in MnCl_2_ group was significantly higher than that in MnSO_4_ and Met-Mn groups at the Mn level of 50–70 mg/kg (*p* < 0.05, Table 6). Mn type only had significant effect on acetate (*p* = 0.01), propionate (*p* < 0.001), butyrate (*p* = 0.023), and total VFAs (*p* = 0.014) levels (Table 6). The acetate and total VFAs levels of Met-Mn group were higher than those of the other two groups (*p* < 0.05); the propionate level of Met-Mn group was higher than that of MnCl_2_ group (*p* < 0.05) but had no significant difference with that of MnSO_4_ group (*p* > 0.05, Table 6). The butyrate level of MnCl_2_ group was higher than that of MnSO_4_ group (*p* < 0.05) but had no significant difference with that of Met-Mn group (*p* < 0.05, Table 6). Mn level had no significant effects on acetate (*p* = 0.075) and propionate (*p* = 0.052), isobutyrate (*p* = 0.687), butyrate (*p* = 0.606), isovalerate (*p* = 0.639), valerate (*p* = 0.569), and total VFAs (*p* = 0.163) levels (Table 6).

**Table 6:** *In vitro* total volatile fatty acids (VFAs) and individual VFAs and acetate/propionate from substrate with added manganese (Mn) from different sources at different levels^1^.

Mn Source	Mn (mg/kg DM)^2^	Total VFA (mmol/L)	Individual VFAs (mmol/L)						A:P ratio
			Acetate	Propionate	Isobutyrate	Butyrate	Isovalerate	Valerate	
MnSO_4_	35 mg/kg	72.4 ± 9.9	40.6 ± 2.3	22.1 ± 5.2	0.5 ± 0.1	7.2 ± 1.7	1.3 ± 0.1	0.8 ± 0.2	1.9 ± 0.4
	40 mg/kg	64.8 ± 10.9	36.2 ± 4.5	19.8 ± 4.3	0.4 ± 0.1	6.6 ± 1.7	1.2 ± 0.1	0.7 ± 0.2	1.9 ± 0.2
	50 mg/kg	70.7 ± 11.9	39.2 ± 5.3	21.5 ± 4.6	0.5 ± 0.1	7.6 ± 1.8	1.2 ± 0.1	0.7 ± 0.2	1.9 ± 0.2 ^B^
	60 mg/kg	62.9 ± 3.8	36.2 ± 2.7	18.6 ± 1.5	0.4 ± 0.0	6.1 ± 0.3	1.0 ± 0.0	0.6 ± 0.1	2.0 ± 0.2 ^B^
	70 mg/kg	68.4 ± 9.1	37.6 ± 4.1	21.2 ± 3.3	0.5 ± 0.1	7.2 ± 1.3	1.2 ± 0.1	0.7 ± 0.1	1.8 ± 0.1 ^B^
*p*-value	Treat	–	–	–	–	–	–	–	0.918
	Linear	–	–	–	–	–	–	–	0.763
	Quadratic	–	–	–	–	–	–	–	0.746
MnCl_2_	35 mg/kg	68.9 ± 9.6	40.1 ± 2.7	19.3 ± 4.6	0.4 ± 0.1	7.3 ± 1.9	1.1 ± 0.1	0.7 ± 0.2	2.1 ± 0.4 ^b^
	40 mg/kg	76.3 ± 22.1	42.1 ± 9.7	22.8 ± 8.1	0.5 ± 0.2	8.7 ± 3.3	1.4 ± 0.2	0.8 ± 0.4	1.9 ± 0.3 ^b^
	50 mg/kg	62.4 ± 20.9	38.9 ± 12.9	12.7 ± 3.7	0.4 ± 0.2	8.6 ± 4.0	1.2 ± 0.2	0.7 ± 0.4	3.1 ± 0.7 ^aA^
	60 mg/kg	53.6 ± 3.2	29.5 ± 1.1	11.3 ± 1.0	0.4 ± 0.1	10.1 ± 0.9	1.4 ± 0.1	0.9 ± 0.1	2.6 ± 0.1 ^abA^
	70 mg/kg	59.1 ± 8.2	34.0 ± 5.3	11.4 ± 2.5	0.5 ± 0.2	10.5 ± 1	1.8 ± 0.2	0.9 ± 0.2	3.0 ± 0.5 ^aA^
*p*-value	Treat	–	–	–	–	–	–	–	0.026
	Linear	–	–	–	–	–	–	–	0.010
	Quadratic	–	–	–	–	–	–	–	0.700
Mn-Met	35 mg/kg	68.2 ± 17.1	39.2 ± 7.1	20.3 ± 7.4	0.3 ± 0.1	6.9 ± 2.4	0.8 ± 0.1	0.7 ± 0.3	2.1 ± 0.6
	40 mg/kg	96.2 ± 17.7	54.3 ± 9.3	29.2 ± 5.9	0.5 ± 0.1	10.0 ± 2.2	1.2 ± 0.1	1.1 ± 0.2	1.9 ± 0.1
	50 mg/kg	78.7 ± 8.9	46.2 ± 9.2	22.6 ± 2.1	0.4 ± 0.0	7.7 ± 0.8	0.9 ± 0.0	0.8 ± 0.1	2.1 ± 0.5 ^B^
	60 mg/kg	82.6 ± 12.5	45.6 ± 5.4	25.1 ± 4.9	0.5 ± 0.1	9.2 ± 2.1	1.2 ± 0.1	1.0 ± 0.2	1.8 ± 0.2 ^B^
	70 mg/kg	65.7 ± 8.2	35.7 ± 4.0	20.4 ± 3.0	0.4 ± 0.1	7.3 ± 1.0	1.0 ± 0.1	0.8 ± 0.1	1.8 ± 0.1 ^B^
*p*-value	Treat	–	–	–	–	–	–	–	0.794
	Linear	–	–	–	–	–	–	–	0.372
	Quadratic	–	–	–	–	–	–	–	0.797
Source	MnSO_4_	67.8 ± 8.9^B^	38 ± 0.3.8^B^	20.6 ± 3.6^A^	0.4 ± 0.1	6.9 ± 1.3 ^B^	1.2 ± 0.3	0.7 ± 0.2	1.9 ± 0.2
	MnCl_2_	64.1 ± 14.9^B^	36.9 ± 8.0^B^	15.5 ± 6.3^B^	0.5 ± 0.1	9.0 ± 2.4 ^A^	1.4 ± 0.5	0.8 ± 0.3	2.6 ± 0.6
	Mn-Met	78.3 ± 16.1^A^	44.2 ± 9.0^A^	23.5 ± 5.5^A^	0.4 ± 0.1	8.2 ± 1.9 ^AB^	1.0 ± 0.2	0.9 ± 0.2	1.9 ± 0.3
Level	35 mg/kg	69.9 ± 11.1	40.0 ± 4.0	20.6 ± 5.2	0.4 ± 0.1	7.1 ± 1.8	1.1 ± 0.3	0.7 ± 0.2	2.0 ± 0.4
	40 mg/kg	79.1 ± 20.5	44.2 ± 10.7	23.9 ± 6.9	0.5 ± 0.1	8.4 ± 2.6	1.2 ± 0.3	0.9 ± 0.3	1.9 ± 0.2
	50 mg/kg	70.6 ± 14.6	41.4 ± 9.1	18.9 ± 5.7	0.4 ± 0.1	7.9 ± 2.3	1.1 ± 0.4	0.7 ± 0.2	2.3 ± 0.7
	60 mg/kg	66.3 ± 14.5	37.1 ± 7.6	18.3 ± 6.5	0.4 ± 0.1	8.5 ± 2.1	1.2 ± 0.3	0.8 ± 0.2	2.1 ± 0.4
	70 mg/kg	64.4 ± 8.4	35.8 ± 4.2	17.7 ± 5.4	0.5 ± 0.1	8.3 ± 1.9	1.3 ± 0.5	0.8 ± 0.2	2.2 ± 0.7
*p-*value	Linear	–	–	–	–	–	–	–	–
	Quadratic	–	–	–	–	–	–	–	–
*p*-value	Source	0.014	0.010	0.000	0.716	0.023	0.056	0.100	–
	Level	0.163	0.075	0.052	0.687	0.606	0.639	0.569	–
	S × L^3^	0.257	0.153	0.122	0.650	0.428	0.592	0.656	0.033

1 Different lowercase letters indicate significant differences in the means of each variable between different Mn levels at the same Mn types (*p* < 0.05), and different uppercase letters indicate significant differences in the means of each variable between different Mn types at the same Mn level (*P* < 0.05).

2 Includes added Mn and Mn in substrate.

3 S × L: Source × Level interaction.

### 3.5. NH_3_-N and MCP levels in fermentation fluid *in vitro*

Mn type (*p* < 0.001) had a significant effect on NH_3_-N concentration while Mn level (*p* = 0.821) and Mn level & type interaction (*p* = 0.266) had no significant effect on it (Table 7). For different Mn types, NH_3_-N concentration of Met-Mn group was significantly higher (*p* < 0.05) than those of MnSO_4_ and MnCl_2_ groups (Table 7). Overview, Mn level & type interaction (*p* < 0.001) had a significant effect on MCP content (Table 7). With the increasing of Mn level, when MnSO_4_ or Met-Mn was used as a Mn source, MCP content showed a quadratic change trend (*p* < 0.001) which increased firstly and then decreased, reaching the highest values at 40 mg/kg and 50 mg/kg Mn levels; when MnCl_2_ was used as a Mn source, MCP content linearly increased (*p* < 0.05) and peaked at 70 mg/kg Mn level group, which was significantly higher than those of 35–50 mg/kg Mn level groups (*p* < 0.05) but was not significantly higher (*p* > 0.05) than that of 60 mg/kg Mn level group (Table 7). For different Mn types, MCP contents of Met-Mn group were significantly higher than those of MnCl_2_ groups at 40–50 mg/kg (*p* < 0.05) Mn levels and were significantly higher than those of MnSO_4_ groups at 40–70 mg/kg (*p* < 0.05) Mn levels (Table 7). Taken together, Mn-Met was the optimal Mn type and 40–50 mg/kg was the optimal Mn level for MCP content in yak’s rumen fermentation *in vitro*.

**Table 7:** *In vitro* total volatile fatty acids (VFAs) and individual VFAs and acetate/propionate ratio from substrate with added manganese (Mn) from different sources at different levels^1^.

Mn Source	Mn (mg/kg DM)^2^	NH_3_-N concentration	Microbial protein
MnSO_4_	35 mg/kg	10.4 ± 0.8	2.0 ± 0.4^bB^
	40 mg/kg	9.9 ± 1.7	3.4 ± 0.4^aB^
	50 mg/kg	10.7 ± 1.1	3.8 ± 0.6^aB^
	60 mg/kg	11.9 ± 1.0	1.3 ± 0.3^cB^
	70 mg/kg	10.8 ± 1.0	0.8 ± 0.1^cC^
*P*-value	Treat	–	0.000
	Linear	–	0.000
	Quadratic	–	0.000
MnCl_2_	35 mg/kg	10.7 ± 1.1	2.8 ± 0.1^cA^
	40 mg/kg	10.0 ± 1.6	3.9 ± 0.2^bB^
	50 mg/kg	10.1 ± 1.1	3.8 ± 0.3^bB^
	60 mg/kg	10.8 ± 2.1	4.1 ± 0.2^abA^
	70 mg/kg	10.1 ± 1.7	4.5 ± 0.2^aA^
*P*-value	Treat	–	0.000
	Linear	–	0.000
	Quadratic	–	0.027
Mn-Met	35 mg/kg	12.4 ± 0.2	1.4 ± 0.5^cB^
	40 mg/kg	13.7 ± 1.2	5.8 ± 0.5^aA^
	50 mg/kg	12.5 ± 0.4	5.5 ± 0.5^aA^
	60 mg/kg	11.7 ± 0.6	4.0 ± 0.5^bA^
	70 mg/kg	11.5 ± 0.3	3.8 ± 0.5^bB^
*P*-value	Treat	–	0.000
	Linear	–	0.010
	Quadratic	–	0.000
Source	MnSO_4_	10.7 ± 1.2^B^	2.3 ± 1.3
	MnCl_2_	10.3 ± 1.4^B^	3.8 ± 0.6
	Mn-Met	12.4 ± 1.0^A^	4.1 ± 1.7
Level	35 mg/kg	11.2 ± 1.1	2.1 ± 0.7
	40 mg/kg	11.2 ± 2.3	4.4 ± 1.1
	50 mg/kg	11.1 ± 1.3	4.4 ± 0.9
	60 mg/kg	11.5 ± 1.3	3.1 ± 1.4
	70 mg/kg	10.8 ± 1.2	3.0 ± 1.3
*P*-value	Linear	–	–
	Quadratic	–	–
*P*-value	Source	0.000	–
	Level	0.821	–
	S × L^3^	0.266	0.000

1 Different lowercase letters indicate significant differences in the means of each variable between different Mn levels at the same Mn types (*P* < 0.05), and different uppercase letters indicate significant differences in the means of each variable between different Mn types at the same Mn level (*p* < 0.05).

2 Includes added Mn and Mn in substrate.

3 S × L: Source × Level interaction.

## 4. Discussion

Rumen is an important fermenter of ruminants and mainly inhabited by microorganisms such as bacteria, fungi, and protozoa. Microorganisms degrade the feed entering the rumen (34) to synthesize VFAs and MCP (35) and provide energy and protein for animals. Rumen efficiency directly affects fermentation end products (25). Gases are produced when substrate carbohydrates and proteins are fermented into VFAs and ammonia. Total gas production has application value in evaluating the nutritional value of feed chemical components and reflecting the formation of short-chain fatty acids and the growth of microbial biomass synthesis (36). In this study, using MnCl_2_ as Mn source and 50 mg/kg Mn level were the most benefit for total gas production. Methane emission is a waste of energy, and in cattle up to 10% of dietary energy is lost through methane emission (37). In this study, we found that neither Mn level, Mn type, nor Mn level & type interaction had significant effect on methane yield.

Rumen pH is an important indicator of rumen environmental stability, and its normal level is between 5.5 and 7.5 (38). Rumen pH affects the growth and proliferation of microorganisms, and thus regulates rumen VFAs production (39). In turn, the production of VFAs and other organic acids such as lactate will also reduce rumen pH (40, 41). It has been reported that the optimal pH for maintaining normal cellulose decomposition is between 6.2 and 7.2 (42). When pH is lower than 6.2, cellulose decomposition bacteria are inhibited (43). The pH of Mn-Met group was significantly lower than that of MnCl_2_ group, which probably caused by the high levels of VFAs in Mn-Met groups (Table 6). Further, the pH of Mn-Met group might be more suitable for microbial biomass and enzyme activities for fiber, starch, and protein breakdown. Trace minerals such as Mn can promote the growth of microorganisms that degrade rumen cellulose, thus improving the fermentability and utilization of organic matter (especially hemicellulose) (17). *In vitro* experiments, the digestion of rumen cellulose was obviously low without Mn supplementation (44), while the digestion rate of rumen cellulose was increased with Mn supplementation (5–30 μg/mL) (45). However, high doses of inorganic Mn (182.7 mg/kg) or organic Mn (184 mg/kg) supplementation decreased α-amylase activity (46). In the current study, we found Mn-Met was the optimal Mn type and 50 mg/kg was the optimal Mn level for digestive enzyme activities in yak’s rumen fermentation. High digestive enzyme activities might improve the digestibility of feed in the process of fermentation. DMD is one of the best methods to estimate the digestibility of feed (47). Mn supplementation improved DMD in rumen fermentation (17, 45, 48). In this study, DMD was increased firstly and then decreased with the increasing of Mn level and DMD of Mn-Met group was higher than that of MnSO_4_ and MnCl_2_ groups (Table 4). The increase in nutrient digestibility related to the addition of Mn might be due to the improvement of metabolic activity of rumen microorganisms or the changes of rumen microbial population (15). Therefore, Mn-Met was the optimal Mn type and 50 mg/kg was the optimal Mn level for feed breakdown in yak’s rumen fermentation.

VFAs are mainly produced by microbial degradation of carbohydrates in the rumen (49) and provide up to 70% of the energy requirements of ruminants (50). Results showed that Mn supplementation had effects on the levels of acetate, propionate, butyrate, total VFAs, and the ratio of acetate to propionate. Acetate is not only used to supply energy through oxidation in tricarboxylic acid cycle but also used to synthesize fatty acids (51). Propionate is the main precursor of gluconeogenesis, while butyrate is mainly converted into β-hydroxybutyrate to provide energy for ruminants (52). Acetate is produced by fiber degradation, while propionate and butyrate are mainly produced by starch degradation (53, 54). Lower acetate/propionate can provide the body with faster and higher energy, which is conducive to improving the growth performance of animals (55). In this study, compared with MnSO_4_ and MnCl_2_ groups, VFAs levels were higher in Met-Mn group, while acetate/propionate value were lower at 50–70 mg/kg Mn level. The supplementation of Met-Mn in the diets of cows and buffalo increased the concentration of total VFAs concentrations (56, 57), which were consistent with our results. Taken together, Mn-Met was the optimal Mn type for yak’s rumen fermentation in terms of VFA production.

Rumen microbiota can degrade the proteins and non-protein substances in the feed into oligopeptides, amino acids, and ammonia, which are then converted into MCP with the help of energy generated by feed fermentation (58). NH_3_-N concentration is the key factor for efficient synthesis of MCP (59), so NH_3_-N concentration is widely used to evaluate essential nitrogen source (25). Low NH_3_-N concentration will inhibit microbial growth and productivity (60), limit rumen fermentation, and ultimately affect rumen ecology (25). In this study, we found that the concentration of ammonia nitrogen in Mn-Met group was higher than those in MnSO_4_ and MnCl_2_ groups. MCP synthesis is affected by energy substance, protein content, and the number and activity of rumen microorganisms (61, 62). We found MCP content increased firstly and then decreased subsequently with the increasing of Mn levels when MnSO_4_ or Mn-Met was used as a Mn source. Further, MCP contents of Mn-Met groups were higher than MnSO_4_ and MnCl_2_ groups at 40–50 mg/kg Mn levels (Table 7). Therefore, 40–50 mg/kg is the optimal Mn levels and Mn-Met is the optimal Mn type for yak’s rumen fermentation in terms of NH_3_-N production and MCP synthesis.

Studies found dietary Mn requirement of sheep was about 20 mg/kg in growing and finishing period (63), Mn requirement of sika deer was 80 mg/kg in terms of feed digestibility (64), and the recommended concentration for breeding cattle is 40 mg/kg Mn (19). In this study, it was found that 40–50 mg/kg Mn level was the most beneficial to yak’s rumen fermentation *in vitro*. The relative utilization rate of different types of Mn in digestive tract of ruminants was different (65). Trace elements in organic form are more beneficial for animals to absorb than inorganic form (66, 67). In a lamb experiment, it was found that the relative bioavailability of Mn-Met was 120% of MnSO_4_ (68). This study also found that organic form Mn-Met is more suitable as Mn source for yak’s rumen fermentation *in vitro*.

## 5. Conclusion

Rumen fermentation *in vitro* of growing yaks was affected by Mn source and Mn level. DMD, digestive enzyme activities, and MCP content in the fermented fluid all showed relatively high values at 40–50 mg/kg Mn levels; further, amylase activity, VFAs levels, NH_3_-N concentration, and MCP content in Mn-Met group were mostly better than those of MnSO_4_ and MnCl_2_ groups. Therefore, Mn-Met was the optimal Mn source and 40–50 mg/kg was the optimal Mn level for yak’s rumen fermentation.

## Data availability statement

The original contributions presented in the study are included in the article/supplementary material, further inquiries can be directed to the corresponding authors.

## Ethics statement

The animal study was reviewed and approved by Professional Committee of Animal Use, Academy of Animal Husbandry and Veterinary Medicine in Qinghai University (QHU20150301).

## Author contributions

YX, LH, and SL: conceptualization and supervision. YX: methodology. HL, PL, and YX: writing—original draft preparation and validation. HL and PL: visualization. YX, HL, PL, and LH: investigation. HL, PL, YX, XZ, BB, JC, ZZ, and CS: formal analysis. YX and LH: resources, data curation, writing—review and editing, project administration, and funding acquisition. All authors contributed to the article and approved the submitted version.

## Funding

This research was funded by the The Open Project of State Key Laboratory of Plateau Ecology and Agriculture (2023-KF-04), AAU Introduction of High-level Talent Funds (RC392107), National Natural Science Foundation of China (32060766), Qinghai Province Key R&D and Transformation Program (2021-NK-126), Key Laboratory of Plateau Grazing Animal Nutrition and Feed Science of Qinghai Province (2022-ZJ-Y17), Top Talent project of “Kunlun Talents–High-level Innovation and Entrepreneurship Talents” in Qinghai Province.

## Conflict of interest

HL, CS, and YX were employed by Qinghai Pure Yak Biotechnology Co., LTD.

The remaining authors declare that the research was conducted in the absence of any commercial or financial relationships that could be construed as a potential conflict of interest.

## Publisher’s note

All claims expressed in this article are solely those of the authors and do not necessarily represent those of their affiliated organizations, or those of the publisher, the editors and the reviewers. Any product that may be evaluated in this article, or claim that may be made by its manufacturer, is not guaranteed or endorsed by the publisher.

## References

[1] Joshi S Shrestha L Bisht N Wu N Ismail M Dorji T. Ethnic and cultural diversity amongst yak herding communities in the Asian highlands. Sustainability. (2020) 12:957. doi: 10.3390/su12030957

[2] Degen AA Kam M Pandey SB Upreti CR Pandey S Regmi P. Transhumant pastoralism in yak production in the lower Mustang District of Nepal. Nomadic Peoples. (2007) 11:57–85. doi: 10.3167/np.2007.110204

[3] Ma L Xu S Liu H Xu T Zhang X. Yak rumen microbial diversity at different forage growth stages of an alpine meadow on the Qinghai-Tibet plateau. PeerJ. (2019) 7:e7645. doi: 10.7717/peerj.7645, PMID: 31579584 PMC6754979

[4] Xu T Xu S Hu L Zhao N Liu Z Ma L. Effect of dietary types on feed intakes, growth performance and economic benefit in Tibetan sheep and yaks on the Qinghai-Tibet plateau during cold season. PLoS One. (2017) 12:e0169187. doi: 10.1371/journal.pone.0169187, PMID: 28056054 PMC5215856

[5] Yoshihara Y Mizuno H Ogura S Sasaki T Sato S. Increasing the number of plant species in a pasture improves the mineral balance of grazing beef cattle. Anim Feed Sci Technol. (2013) 179:138–43. doi: 10.1016/j.anifeedsci.2012.11.009

[6] Shannon MC Hill GM. Trace mineral supplementation for the intestinal health of young Monogastric animals. Front Vet Sci. (2019) 6:73. doi: 10.3389/fvets.2019.00073, PMID: 30918894 PMC6424858

[7] Jurjanz S Collas C Lastel M-L Godard X Archimède H Rychen G. Evaluation of soil intake by growing creole young bulls in common grazing Systems in Humid Tropical Conditions. Animal. (2017) 11:1363–71. doi: 10.1017/S1751731116002755, PMID: 28069088

[8] Fan Q Wang Z Chang S Peng Z Wanapat M Bowatte S. Relationship of mineral elements in sheep grazing in the Highland agro-ecosystem. Asian Australas J Anim Sci. (2020) 33:44–52. doi: 10.5713/ajas.18.0955, PMID: 31010963 PMC6946991

[9] Xin GS Long RJ Guo XS Irvine J Ding LM Ding LL. Blood mineral status of grazing Tibetan sheep in the northeast of the Qinghai–Tibetan plateau. Livest Sci. (2011) 136:102–7. doi: 10.1016/j.livsci.2010.08.007

[10] Fan Q Wanapat M Hou F. Mineral nutritional status of yaks (*Bos Grunniens*) grazing on the Qinghai-Tibetan plateau. Animals. (2019) 9:468. doi: 10.3390/ani9070468, PMID: 31340454 PMC6680518

[11] Yu Y Mizuno H Yasue H Purevdorj N Ito TY. Nomadic grazing improves the mineral balance of livestock through the intake of diverse plant species. Anim Feed Sci Technol. (2013) 184:80–5. doi: 10.1016/j.anifeedsci.2013.06.007

[12] Pajarillo EAB Lee E Kang D-K. Trace metals and animal health: interplay of the gut microbiota with Iron, manganese, zinc, and copper. Anim Nutr. (2021) 7:750–61. doi: 10.1016/j.aninu.2021.03.005, PMID: 34466679 PMC8379138

[13] Leach R Jr Muenster A-M. Studies on the role of manganese in bone formation: I. effect upon the Mucopolysaccharide content of Chick bone. J Nutr. (1962) 78:51–6. doi: 10.1093/jn/78.1.51 14463214

[14] Leach RM Muenster AM Wien EM. Studies on the role of manganese in bone formation. Ii. Effect upon chondroitin sulfate synthesis in Chick epiphyseal cartilage. Arch Biochem Biophys. (1969) 133:22–8. doi: 10.1016/0003-9861(69)90483-4, PMID: 5810830

[15] Gresakova L Venglovska K Cobanova K. Nutrient digestibility in lambs supplemented with different dietary manganese sources. Livest Sci. (2018) 214:282–7. doi: 10.1016/j.livsci.2018.07.001

[16] Hilal EY Elkhairey M Osman A. The role of zinc, Manganse and copper in rumen metabolism and immune function: a review article. Open J Anim Sci. (2016) 6:304–24. doi: 10.4236/ojas.2016.64035

[17] Tiwari S Jain R Mishra U Mishra O Patel J Rajagopal S. Effect of trace mineral (mineral capsule) supplementation on nutrient utilization and rumen fermentation pattern in Sahiwal cows (*Bos Indicus*). Indian J Anim Sci. (2000) 18:409–22. doi: 10.1016/S0739-7240(00)00059-X

[18] Sonawane S Arora S. Influence of zinc supplementation on rumen microbial protein synthesis in in vitro studies [India]. Indian J Anim Sci. (1976) 46:13–8

[19] Wang S Wang Y Hu Z Chen Z Fleckenstein J Schnug E. Status of Iron, manganese, copper, and zinc of soils and plants and their requirement for ruminants in Inner Mongolia steppes of China. Commun Soil Sci Plant Anal. (2003) 34:655–70. doi: 10.1081/CSS-120018966

[20] Hidiroglou M. Manganese in ruminant nutrition. Can J Anim Sci. (1979) 59:217–36. doi: 10.4141/cjas79-028

[21] Council NR. Nutrient requirements of dairy cattle: 2001. Washington, DC: National Academies Press (2001). 38386771

[22] Chu GM Komori M Hattori R Matsui T. Dietary Phytase increases the true absorption and endogenous fecal excretion of zinc in growing pigs given a corn-soybean meal based diet. Anim Sci J. (2009) 80:46–51. doi: 10.1111/j.1740-0929.2008.00595.x 20163467

[23] Spears JW. Trace mineral bioavailability in ruminants. J Nutr. (2003) 133:1506S–9S. doi: 10.1093/jn/133.5.1506s 12730454

[24] National Academies of Sciences E, Medicine. Nutrient requirements of beef cattle. Washington, DC: The National Academies Press (2016).

[25] Wanapat M. Rumen manipulation to increase the efficient use of local feed resources and productivity of ruminants in the tropics. Asian Australas J Anim Sci. (2000) 13:59–67.

[26] Ministry of Agriculture and rural Affairs of the People’s republic of China. Feeding standard of beef cattle (NY/T815-2004). Beijing, China: China Agriculture Press (2004) (In Chinese).

[27] Hu LH. Recent advances in yak nutrition. Xining, China: Qinghai People’s Press (1997) (In Chinese).

[28] GB/T13885-2003. Animal feeding stuffs-determination of contents of calcium, copper, Iron, magnesium, manganese potassium, sodium and zinc-method Ssing atomic absorption spectrometry. Geneva, Switzerland: ISO International Standard (2003).

[29] Liu J-F Feng Y-D Jiang G-B. Identical flow injection spectrophotometric manifold for determination of protein, phosphorus, calcium, chloride, copper, manganese, Iron, and zinc in feeds or premixes. J AOAC Int. (2001) 84:1179–86. doi: 10.1093/jaoac/84.4.1179 11501921

[30] Menke KH Raab L Salewski A Steingass H Schneider W. The estimation of the digestibility and Metabolizable energy content of ruminant Feedingstuffs from the gas production when they are incubated with rumen liquor in vitro. J Agric Sci. (1979) 93:217–22. doi: 10.1017/S0021859600086305

[31] Menke KH. Estimation of the energetic feed value obtained from chemical analysis and in vitro gas production using rumen fluid. Anim Res Dev. (1988) 28:7–55.

[32] Feng ZC Gao M. Improvement of the method for determination of Ammonia nitrogen content in rumen fluid by colorimetry. Anim husb feed sci. (2010) 31:37. doi: 10.16003/j.cnki.issn1672-5190.2010.z1.027. (In Chinese)

[33] Zhao X Hao L Xue Y Degen A Liu S. Effect of source and level of dietary supplementary copper on in vitro rumen fermentation in growing yaks. Fermentation. (2022) 8:693. doi: 10.3390/fermentation8120693

[34] Zhou Z Fang L Meng Q Li S Chai S Liu S. Assessment of ruminal bacterial and archaeal community structure in yak (*Bos Grunniens*). Front Microbiol. (2017) 8:179. doi: 10.3389/fmicb.2017.00179, PMID: 28223980 PMC5293774

[35] Bergmann GT. Microbial community composition along the digestive tract in forage-and grain-fed Bison. BMC Vet Res. (2017) 13:1–9. doi: 10.1186/s12917-017-1161-x, PMID: 28818110 PMC5561592

[36] Getachew G Blümmel M Makkar H Becker K. In vitro gas measuring techniques for assessment of nutritional quality of feeds: a review. Anim Feed Sci Technol. (1998) 72:261–81. doi: 10.1016/s0377-8401(97)00189-2

[37] Blaxter KL. The energy metabolism of ruminants. Energy Metabolism Ruminants. (1962) 139. doi: 10.1136/vr.139.14.350-a

[38] Melo LQ Costa SF Lopes F Guerreiro MC Pereira MN. Rumen Morphometrics and the effect of Digesta Ph and volume on volatile fatty acid absorption. J Anim Sci. (2013) 91:1775–83. doi: 10.2527/jas.2011-4999 23345561

[39] Wales WJ Kolver ES Thorne PL Egan AR. Diurnal variation in ruminal Ph on the digestibility of highly digestible perennial ryegrass during continuous culture fermentation. J Dairy Sci. (2004) 87:1864–71. doi: 10.3168/jds.S0022-0302(04)73344-5 15453503

[40] Geishauser T Linhart N Neidl A Reimann A. Factors associated with ruminal Ph at herd level. J Dairy Sci. (2012) 95:4556–67. doi: 10.3168/jds.2012-5380 22818470

[41] Eun J-S Beauchemin K. Effects of a proteolytic feed enzyme on intake, digestion, ruminal fermentation, and Milk production. J Dairy Sci. (2005) 88:2140–53. doi: 10.3168/jds.s0022-0302(05)72890-3, PMID: 15905444

[42] Van Soest PJ. Nutritional ecology of the ruminant. Ithaca, NY: Cornell University Press (1994).

[43] Grant RH Mertens DR. Influence of buffer Ph and raw corn starch addition on in vitro Fiber digestion kinetics. J Dairy Sci. (1992) 75:2762–8. doi: 10.3168/jds.S0022-0302(92)78039-4, PMID: 1331215

[44] Chamberlain C Burroughs W. Effect of fluoride, magnesium and manganese ions on in vitro cellulose digestion by rumen microorganisms. J Anim Sci. (1962) 21:428–32. doi: 10.1103/PhysRevC.79.054317

[45] Martinez A Church D. Effect of various mineral elements on in vitro rumen cellulose digestion. J Anim Sci. (1970) 31:982–90. doi: 10.2527/jas1970.315982x, PMID: 5481274

[46] Kišidayová S Pristaš P Zimovčáková M Blanár Wencelová M Homol’ová L Mihaliková K. The effects of high dose of two manganese supplements (organic and inorganic) on the rumen microbial ecosystem. PLoS One. (2018) 13:e0191158. doi: 10.1371/journal.pone.0191158, PMID: 29324899 PMC5764370

[47] Weiss WP. Estimation of digestibility of forages by laboratory methods. Forage Quality Eval Utilization. (1994):644–81. doi: 10.2134/1994.foragequality.c16

[48] Arelovich H Owens F Horn G Vizcarra J. Effects of supplemental zinc and manganese on ruminal fermentation, forage intake, and digestion by cattle fed prairie Hay and urea. J Anim Sci. (2000) 78:2972–9. doi: 10.2527/2000.78112972x, PMID: 11063324

[49] He Y Wang H Yu Z Niu W Qiu Q Su H. Effects of the gender differences in cattle rumen fermentation on anaerobic fermentation of wheat straw. J Clean Prod. (2018) 205:845–53. doi: 10.1016/j.jclepro.2018.09.156

[50] Bergman E. Energy contributions of volatile fatty acids from the gastrointestinal tract in various species. Physiol Rev. (1990) 70:567–90. doi: 10.1152/physrev.1990.70.2.567, PMID: 2181501

[51] Bach A Yoon IK Stern MD Jung HG Chester-Jones H. Effects of type of carbohydrate supplementation to lush pasture on microbial fermentation in continuous culture. J Dairy Sci. (1999) 82:153–60. doi: 10.3168/jds.S0022-0302(99)75219-7, PMID: 10022017

[52] Phillips RW Black AL. The effect of volatile fatty acids on plasma glucose concentration. Comp Biochem Physiol. (1966) 18:527,IN3,33–32,IN3,36. doi: 10.1016/0010-406X(66)90237-4 5967680

[53] Janssen PH. Influence of hydrogen on rumen methane formation and fermentation balances through microbial growth kinetics and fermentation thermodynamics. Anim Feed Sci Technol. (2010) 160:1–22. doi: 10.1016/j.anifeedsci.2010.07.002

[54] Giger-Reverdin S Rigalma K Desnoyers M Sauvant D Duvaux-Ponter C. Effect of concentrate level on feeding behavior and rumen and blood parameters in dairy goats: relationships between behavioral and physiological parameters and effect of between-animal variability. J Dairy Sci. (2014) 97:4367–78. doi: 10.3168/jds.2013-7383, PMID: 24952476

[55] Ma J Mujtaba Shah A Shao Y Wang Z Zou H Hu R. Effects of yeast Cell Wall on the growth performance, ruminal fermentation, and microbial Community of Weaned Calves. Livest Sci. (2020) 239:104170. doi: 10.1016/j.livsci.2020.104170

[56] El-Esawy GS Riad W El-Dein AM Ali M Gaafar H. Effect of supplementary zinc and manganese methionine chelates on productive and reproductive performance of dairy Friesian cows. Archiva Zootechnica. (2017) 96:289–302. doi: 10.21608/ejar.2018.132846

[57] Shakweer I Mustafa G Ahmed G Ismail M. Effect of zinc or/and manganese methionine supplements on performance of lactating buffaloes. J Anim Poul Prod. (2010) 1:589–602. doi: 10.21608/jappmu.2010.86271

[58] Owens FN Basalan M. Ruminal Fermentation. Rumenology. (2016):63–102. doi: 10.1007/978-3-319-30533-2_3

[59] Stern M Hoover WH. Methods for determining and factors affecting rumen microbial protein synthesis: a review. J Anim Sci. (1979) 49:1590–603. doi: 10.2527/jas1979.4961590x

[60] Satter L Slyter L. Effect of Ammonia concentration on rumen microbial protein production in vitro. Br J Nutr. (1974) 32:199–208. doi: 10.1079/bjn19740073 4472574

[61] Davis C. Some aspects of feeding high producing dairy cows. J Dairy Sci. (1980) 63:873–85. doi: 10.3168/jds.S0022-0302(80)83021-9

[62] Clark J Davis C. Future improvement of Milk production: potential for nutritional improvement. J Anim Sci. (1983) 57:750–64. doi: 10.2527/jas1983.573750x 6355043

[63] Rc N. Nutrient requirements of sheep. Natl Acad Sci. (1975) 2:73–87. doi: 10.4081/ijas.2003.73

[64] Bao K Wang K Wang X Zhang T Liu H Li G. Effects of dietary manganese supplementation on nutrient digestibility and production performance in male sika deer (*Cervus Nippon*). Anim Sci J. (2017) 88:463–7. doi: 10.1111/asj.12657, PMID: 27481564

[65] Spears JW. Organic trace minerals in ruminant nutrition. Anim Feed Sci Technol. (1996) 58:151–63. doi: 10.1016/0377-8401(95)00881-0

[66] Gresakova L Venglovska K Cobanova K. Dietary manganese source does not affect Mn, Zn and cu tissue deposition and the activity of manganese-containing enzymes in lambs. J Trace Elem Med Biol. (2016) 38:138–43. doi: 10.1016/j.jtemb.2016.05.003 27267351

[67] Wright C Spears J Webb K Jr. Uptake of zinc from zinc sulfate and zinc Proteinate by ovine ruminal and Omasal epithelia. J Anim Sci. (2008) 86:1357–63. doi: 10.2527/jas.2006-650, PMID: 18310488

[68] Henry P Ammerman C Littell R. Relative bioavailability of manganese from a manganese-methionine complex and inorganic sources for ruminants. J Dairy Sci. (1992) 75:3473–8. doi: 10.3168/jds.s0022-0302(92)78123-5, PMID: 1474213

